# Transportation of Wounded

**Published:** 1918-09

**Authors:** 


**Transportation of Wounded.** By Médecin-Major Proust.

It must always be borne in mind, the speaker said, that repeated handling and movement are extremely undesirable for wounded men. The ideal would be immediate and uninterrupted transport to the desired destination once examination and designation has been made.

We will now consider the best division of labor for efficient clearance of wounded in the three main centers of operation which are: the group of stretcher bearers, the group of army corps ambulances, and the evacuation hospital. These are the three main divisions of the sieve through which the wounded pass with a minimum number of examinations and dressings.

I. The Group of Brancardiers (stretcher bearers) usually known s G. B. D. are at the point of embarcation of the sanitary transports, and distinguish between (*a*) the absolutely untransportable cases and (*b*) the fracture cases.

The absolutely untransportable cases are sent to a neighbouring dressing station reserved exclusively for such cases. Among these should be counted all who are wearing tourniquets.

The fractures should be thoroughly and carefully placed in splints which, once adjusted, enable the patients to stand the journey to the special army hospitals for fractures.

Outside of these two classes there remain the great majority of the wounded who demand, for proper classification, an installation and a personnel which the G. B. D. cannot provide. The only thing to do, in a time of offensive, is to separate the sitting cases, which can go by camions to some point of embarcation by train, and the lying down cases which are taken to the groups of Army ambulances, fifteen to twenty kilometers back of the front where the real classification and clearing is done under the direction of surgeons.

II. Group of Army Corps Ambulances.

The role of this group is to obtain results which would not be possible in an isolated ambulance, and it constitutes an excellent clearing-house for the wounded. It helps in eliminating any slightly wounded cases that should not be allowed to clog at this point. Then the wounded are examined, new dressings given, and a certain number of cases are set aside for operation ; the number thereof corresponds to the daily operative capacity of the ambulance. These cases are relatively untransportable.

A certain number of reception rooms should be provided at the army corps ambulance for such wounded as are greatly fatigued by the trip. Those of them who have been examined and whose dressings have been remade under aseptic conditions and who are now capable of going further, are sent back to the H. O. E. or Hospital of Evacuation. The only exceptions to this are those fracture cases which by mistake may be found in the Army corps ambulance. These are sent immediately to the special Hospital of fracture service of the Army. The speaker insisted upon this point — that the wounded admitted to the Army ambulance, who should receive only a new dressing and an injection of anti-tetanic serum, should under no circumstances be allowed to remain there. Any head surgeon who allows wounded to accumulate there does not understand his duty, which is quickly to forward all cases for which he cannot give the required treatment.

The difficulty here is that the means of transportation from the front are more numerous than the means of transporting them to the rear, thereby causing an accumulation in the Army Ambulance. For this reason there should be a system of automobile transports with a large reserve of cars which could immediately relieve the congestion where necessary. The wounded who have left the group of army ambulances are then sent on to hospital of evacuation.

III. The role of the H. O. E. is to distinguish between the cases needing immediate operation in one of its affiliated hospitals, and those capable of being sent on by train. During offensives, it becomes necessary to make up trains of cases needing operation, whose condition, however, seems good enough to endure the journey. During quiet periods the affiliated hospitals should operate on all cases, and the H. O. E. should evacuate only those cases which have been operated upon and which have become capable of evacuation. The operated cases and the fractures are evacuated then to specialized center for continued treatment.

To return to the large group of the slightly wounded who were put in camions at the G. B. D. Among them are very slight wounds, which, during the fighting in fixed positions, would be taken care of at some special post for the slightly wounded. The operation of these posts becomes difficult during this war of movement, and it often happens that these wounded have to be evacuated. It would be advisable to have at the point where the railroad leaves the front zone, a large sanitary station for the slightly wounded, which would assist rapid recovery and avoid congestion in the front zone.

The G. B. D. simply eliminates the absolutely untransportable cases which it sends to an advanced ambulance, and the fractures which it puts in splints and sends to the center of fractures. The group of ambulances examines all the wounded. This is the real sorting station, and it sends all cases which do not require immediate operation to its affiliated hospitals. Only in times of offensive does it evacuate those needing operations. The slightly wounded, taken by camion, are, during an offensive, evacuated by train to some base hospital along the railroad line of the hospital train service of the front.

During a period of intense military activity, certain precautionary measures are taken to meet the flood of wounded, so that there shall be no blocking at any point. The first halt comes with the recording of the wounded upon their arrival. This should be met with an increased number of the clerical force, or by a simplification of the required records. There should also be an increased number of places of embarcation for the trains, the stations being very limited in capacity.

